# Patient reported symptoms, coping and quality of life during somatostatin analogue treatment for metastatic small- intestinal neuroendocrine tumours

**DOI:** 10.1186/s12955-020-01452-7

**Published:** 2020-06-16

**Authors:** Halfdan Sorbye, Liv Sylvi Meyer, Kjersti Elisabeth Mordal, Simen Myhre, Espen Thiis-Evensen

**Affiliations:** 1 Department of Oncology, Haukeland University Hospital, Jonas Lies vei 65, 5021 Bergen, Norway; 2 Department of Clinical Science, University of Bergen, Bergen, Norway; 3 Neuroendocrine Tumour Centre of Excellence, Department of Gastroenterology, Oslo University Hospital, Rikshospitalet, Oslo, Norway; 4 Novartis Norway AS, Oslo, Norway

**Keywords:** Quality of life, Small-intestinal neuroendocrine tumour, Patient reported symptoms, Coping, Somatostatin analogue

## Abstract

**Background:**

Patients with metastatic small-intestinal neuroendocrine tumours (NET) have been shown to have a reduced quality of life compared to the general population and many have disabling symptoms during somatostatin analogue (SSA) treatment. The aim of this prospective study was to document the patient-reported symptoms, coping and quality of life during SSA treatment and to measure patients’ fat-soluble vitamin levels.

**Methods:**

Patients with metastatic small-intestinal NET on treatment with long-acting SSA were included. Data on patient characteristics, blood samples, questionnaires (EORTC-QLQ-C30 and GI.NET-21) and structured patient interviews were collected at inclusion and after 1 year.

**Results:**

Eighty-eight patients were included, 77 (88%) attended 1 year follow-up. Approximately 50% of patients reported symptoms, the most common symptoms at baseline and after 1 year follow-up were diarrhoea, flatulence, fatigue, abdominal discomfort and sore injection lumps. Diarrhoea and fatigue were reported as their main complaint, 23% had > 5 daily episodes of diarrhoea and 59% reported fatigue. However, patients reported a high perceived quality of life, high daily activity, coped with their symptoms and managed their daily life well. Deficiency of vitamin D (27%) and A (13%) were observed.

**Conclusions:**

Patients with metastatic small-intestinal NET on SSA treatment reported a high frequency of symptoms. Minor improvements were seen after 1-year of follow-up, illustrating that many symptoms might be difficult to improve, or may not be recognised by the health service. Patients, however, generally reported a high quality of life. Care for NET patients on SSA treatment should include a regular systematic symptom registration and vitamin measurements.

## Background

The incidence of neuroendocrine tumours (NET) is increasing [1, 2]. NET is classified based on growth rate (Ki-67 estimate) and organ of origin with the small-intestine as the most common primary site [3–5]. Survival for patients with metastatic small-intestinal NET is frequently 10–12 years [6]. In metastatic small-intestinal NET, first-line treatment is usually somatostatin analogues (SSA) which inhibit hormone production and reduce the patient’s flushing and diarrhoea. Octreotide long-acting release (LAR) and lanreotide have also been shown to inhibit tumour growth and prolong progression-free survival/time to progression in patients with small-intestinal NET [7, 8]. SSA is usually continued during other NET targeted treatments as peptide receptor radionuclide therapy [9]. NET patients are therefore frequently on SSA treatment for a considerable number of years. Patients with metastatic small-intestinal NET often have symptoms from their disease. Flushing is the most classic symptom and can be triggered by both physical and mental exertions as well as meals and alcohol. Flushing varies in duration and intensity, from 2 to 5 min up to several hours. Diarrhoea is experienced by many patients and some NET patients may have bowel movements up to 20 times a day and reduced nutritional uptake may occur. Diarrhoea-reducing drugs such as loperamide or opium drops can be tried, but data on the frequency of their use and the benefit the patient experiences are scarce. It might be difficult to separate symptoms from the metastatic disease itself, SSA side-effects or previous surgery. SSA treatment may inhibit excretion of pancreatic enzymes and induce malabsorption which can cause diarrhoea and flatulence. Increased gas, often with a characteristic malodour, is a frequent complaint of patients and can have a substantial social impact. The malabsorption can be treated by supplementation of pancreatic enzymes, however frequently the effect is limited. Surgery, usually intestinal resection with removal of the primary tumour, can give symptoms due to altered intestinal motility, bacterial overgrowth and intestinal adhesions. Patients with NET have been found to have a reduced quality of life with fatigue, tumour-related pain, anxiety and depression [10–12]. A Norwegian study on patients with all types of NET showed a reduced overall health and vitality and reduced capacity to participate in daily activities compared to the general population [13].

In the present study we performed a systematic registration of symptoms, treatment of symptoms and overall well-being of NET patients using SSA at study inclusion and after 1 year. A detailed overview of these aspects could be helpful in designing approaches to improve the quality of life of this patient group. Vitamin measurements were also included as a these patients can develop deficiencies in fat-soluble vitamins [14].

## Methods

### Study design

This was a 1-year prospective observational cohort study performed at the two largest NET centres in Norway. All NET patients in the uptake area are treated at these centres, thus the patients should represent a population-based cohort on SSA treatment. The aim was to evaluate and describe the symptoms, quality of life and fat-soluble vitamin levels in patients with metastatic small-intestinal neuroendocrine tumours. The data were collected during Jan 2014 to Aug 2017.

### Patient population

Inclusion criteria were patients with non-resectable metastatic neuroendocrine tumours with a Ki-67 < 20% (WHO G1-G2) with either a known origin in the small-intestine or a highly probable origin in the small-intestine. Patients had to be on treatment with a long-acting SSA and have a life expectancy of more than 6 months. The study was approved by the regional Ethics Committee and informed consent was obtained from each patient.

### Data collection procedures

The following data were collected from medical records: surgery, disease location, Ki-67 tumour estimate, general clinical condition, medical treatment, co-morbidity, laboratory markers including Chromogranin A (CgA) in blood and 5-hydroxyindoleacetic acid (5-HIAA) in urine, age, gender, occupation and disease status based on last computed tomography (CT) evaluation (stable disease/progression). Blood samples were taken at baseline and at 1-year follow-up to detect possible deficiencies in fat-soluble vitamins (vitamins A, D, E and K).

### Questionnaires and interviews

Patients were asked to respond to standardised and validated quality of life questionnaires, including one general questionnaire and one supplementary NET questionnaire, both developed by the European Organisation for Research and Treatment of Cancer (EORTC QLQ-30, version 3 and QLQ G.I.NET21). The EORTC QLQ.C30 includes 30 questions incorporated in five functional scales: physical, role, cognitive, emotional, and social; nine symptom scales: fatigue, pain, nausea, vomiting, dyspnea, appetite loss, sleep disturbance, constipation, diarrhea; and a global health and quality of life scale [15]. The questionnaire results were converted to 1–100 scale. A higher score on functional scales represented a higher level of function, while a higher score on symptom scales represented a higher level of symptoms. To assess NET specific symptoms the QLQ G.I.NET21 questionnaire was used [16]. This questionnaire contains four single-item assessments relating to muscle/bone pain, sexual function, information/communication function, body image and five scales describing endocrine symptoms, gastrointestinal symptoms, treatment-related symptoms, social function symptoms and disease-related worries on a 1–100 scale with higher scores reflecting more severe symptoms. In addition, a local questionnaire with relevant questions about patient reported symptoms, WHO performance status, impact on daily life and side effects of drug treatment with SSA was included using in part the Birmingham IBS Symptom Questionnaire [17]. Patients were also interviewed by a specialised NET nurse (LSM, KEM) to collect information on symptoms, activity of daily life, injection side effects, effects on dietary habits and use of prior symptom-relieving treatment, both physician and patient initiated.

### Statistical analysis

Given the descriptive purpose and the non-clinically relevant differences from baseline to 1-year follow-up, we decided to avoid unnecessary statistical testing. The results from this study are therefore only presented descriptively. Continuous data are summarised using descriptive statistics and categorical data are presented using absolute frequency and percentage. All available patients and data are included in the descriptive tables. The denominator for percentage calculations is the total number of patients with available data. For variables with missing values, the number of patients with missing values is presented.

## Results

### Patient characteristics and prior treatments

In total 88 patients fulfilled the inclusion criteria and provided data at baseline and 77 at 1-year follow-up. Patients who did not complete 1-year follow-up had either died (7 patients), did not want to participate in the follow-up (2 patients), had been transferred to another hospital (1 patient) or was not able to perform the questionnaires due to dementia (1 patient). Main baseline characteristics are shown in Table 1. A primary small-intestinal NET had been diagnosed in 82% of the patients, 18% had an undiagnosed primary, but suspected to be small-intestinal. More than two-thirds of patients (69%) did not have any weight loss in the preceding year and patients had no major changes in weight during the 1 year follow-up. Based on the last CT evaluation before baseline, 15 patients (17%) had progressive disease at baseline while 73 (83%) had non-progression. At 1-year follow-up evaluation, the patients remaining in the study had overall similar disease characteristics as compared to baseline in terms of performance status, co-morbidity, and CT results (data not shown). Pancreatic enzyme supplements were used by 30% of patients at baseline, whereas loperamide and cholestyramine were used by 23 and 7% respectively. Many patients had prior usage of these treatments. Usage was similar at the 1-year follow-up (Table 1). Only 7% of the patients reported using additional alternative medication (not physician-prescribed) for treatment of NET. The majority of patients in our study were retired (60%). Of the 35 non-retired patients, 15 (43%) were working full- or part-time (Table 1).

**Table 1:** Baseline characteristics of patients on somatostatin analogue treatment for metastatic disease from small-intestinal neuroendocrine tumour

	Baseline ***N*** = 88	1-year follow-up ***N*** = 77
**Males,*n*(%)**	47 (53.4%)	
**Age, median (range)**	65.0 (32–87) years	
**Time since diagnosis, median (range)**	4.82 (0.3–34.3) years	
**Time since first treatment, median (range)**	3.09 (0.2–15.3) years	
**Weight, median (range)**	72.5 (40–130) kg	70.0 (40–132) kg
**Weight change from baseline, median (range)**		0.0 (−15–13) kg
**Weight loss in the last year,*n*(%)**		
**None**	61 (69.3%)	51 (67.1%)^b^
**0–5%**	18 (20.5%)	17 (22.4%)^b^
**6–10%**	7 (8.0%)	6 (7.9%)^b^
**> 10%**	2 (2.3%)	2 (2.6%)^b^
**Occupation,*n*(%)**		
**Retired**	53 (60.2%)	50 (66.7%)^b^
**Non-retired**	35 (39.8%)	25 (33.3%)^b^
*Working (% of non-retired)*	*15 (42.9%)*	*12 (48.0%)*^*b*^
*Not working (% of non-retired)*	*20 (57.1%)*	*13 (52.0%)*^*b*^
**Disease status,*n*(%)**		
**Metastatic mesenteric lymph nodes**	34 (38.6%)	
**Other metastatic lymph nodes**	35 (39.8%)	
**Liver metastases**	78 (88.8%)	
**Lung metastases**	1 (1.1%)	
**Other metastases**	23 (26.1%)	
**Surgery of primary tumour,*n*(%)**	74 (84.1%)	
**Surgery of mesenteric lymph nodes**	42 (49.4%)^b^	
**Surgery of liver metastases**	14 (17.1%)^b^	
**Ki67, median (min-max)**	1.0% (1–15%)	
**Ki67, categorised**		
**0–2%**	60 (68.2%)	
**> 2%**	28 (31.8%)	
**Performance status,*n*(%)**		
**0**	49 (55.7%)	48 (62.3%)
**1**	31 (35.2%)	16 (20.8%)
**2**	7 (8.0%)	11 (14.3%)
**3**	1 (1.1%)	2 (2.6%)
**Smoker,*n*(%)**	11 (12.6%)^b^	
**Prior treatments**		
**Interferon**	22 (25.0%)	4 (5.2%)^c^
**PRRT**	21 (23.9%)	19 (24.7%)^c^
**5-HIAA in urine, above normal,*n*(%)**	42 (79.2%)^**b**^	25 (83.3%)^b^
**CgA in blood, above normal,*n*(%)**	64 (72.7%)	55 (75.3%)^b^
**Pancreatic enzyme supplement,*n*(%)**		
**Current use**	26 (29.5%)	27 (35.5%)^b^
**Prior and current use**	39 (44.3%)	NA
**Loperamide,*n*(%)**		
**Current use**	20 (22.7%)	17 (22.4%)^b^
**Prior and current use**	38 (43.2%)	NA
**Cholestyramine,*n*(%)**		
**Current use**	6 (6.8%)	6 (7.9%)^b^
**Prior and current use**	13 (14.8%)	NA
**Vitamin supplements,*n*(%)**		
**Current use**	59 (67.0%)	64 (83.1%)

*5-HIAA* 5-hydroxyindoleacetic acid, *CgA* Chromogranin A, *ECOG* Eastern Cooperative Oncology Group, *PRRT* peptide receptor radionuclide therapy

### Somatostatin analogue treatment

Octreotide LAR was used by 61% of patients while 39% used lanreotide. The median time since the start of SSA treatment was 3.1 years (range: 0.2–15.3 years). The injections were given at 3-to-4-week intervals for the vast majority of patients (3-week intervals: 35%, 4-week intervals: 56% and 2-week intervals: 9%). The type of SSA treatment and injection intervals remained approximately the same throughout the year of the study (data not shown). Almost all patients reported that the main indication for treatment were to live longer (92%) and reduce symptoms (57%). A vast majority of the patients responded that they had no chance of a cure from the disease (90%).

### Quality of life and symptoms – questionnaires

In the EORTC QLQ-C30 questionnaire, patients reported high mean scores, corresponding to good self-assessed quality of life at baseline for global health status and the five functional scales (physical, role, cognitive, emotional and social functioning) (Table 2). There were only minor improvements at the 1-year follow-up. Based on the symptom scales, diarrhoea and fatigue were the most severe symptoms. In the symptom specific QLQ G.I.NET21 questionnaire, patients reported the highest mean for disease-related worries and social function (Table 3). Results from the single item assessments showed that muscle/bone pain and sexual function were the two areas where patients reported most symptoms. There were only minor improvements at the 1-year follow-up.

**Table 2:** Symptoms in patients on somatostatin analogue treatment for metastatic disease from small-intestinal neuroendocrine tumour EORTC QLQ-C30 values expressed as mean (standard deviation). A higher score (1–100) on functional scales represents a higher level of function, while a higher score (1–100) on symptom scales represents a higher level of symptoms

	Baseline	1-year follow-up
**Functional scores**		
Global health status	72.5 (20.4)	75.5 (18.7)
Physical functioning	80.2 (20.6)	82.0 (19.1)
Role functioning	77.5 (27.9)	78.4 (23.7)
Emotional functioning	83.1 (21.9)	85.4 (17.3)
Cognitive functioning	79.0 (19.0)	81.4 (17.3)
Social functioning	71.4 (25.5)	78.8 (24.6)
Financial difficulties	5.3 (15.1)	5.6 (14.7)
**Symptom scores**		
Fatigue	35.3 (23.0)	34.4 (22.1)
Nausea and vomiting	5.5 (10.3)	4.8 (9.7)
Pain	22.9 (28.2)	19.3 (25.9)
Dyspnoea	25.2 (28.9)	22.5 (25.6)
Insomnia	30.7 (36.5)	25.4 (29.8)
Appetite loss	9.1 (21.9)	7.0 (20.6)
Constipation	14.0 (21.9)	10.5 (19.0)
Diarrhoea	51.5 (32.7)	42.2 (31.7)

**Table 3:** Symptoms in patients on somatostatin analogue treatment for metastatic disease from small-intestinal neuroendocrine tumour. EORTC GI.NET-21, values expressed as mean (standard deviation) Higher scores (1–100) indicate more severe symptoms

	Baseline	1-year follow-up
**Endocrine symptoms**	15.6 (17.5)	13.0 (15.7)
**Gastrointestinal symptoms**	21.2 (15.6)	18.7 (15.5)
**Treatment-related symptoms**	12.0 (14.1)	10.2 (12.1)
**Social functioning**	27.8 (19.5)	25.4 (17.7)
**Disease-related worries**	34.2 (21.5)	30.1 (21.9)
**Muscle/bone pain**	31.4 (32.9)	25.5 (29.1)
**Sexual functioning**	29.2 (36.4)	28.6 (37.8)
**Information/communication**	6.1 (18.6)	5.6 (14.7)
**Body image**	16.7 (28.6)	13.9 (24.4)

### Quality of life and symptoms – nurse interviews

**Fig. 1:**
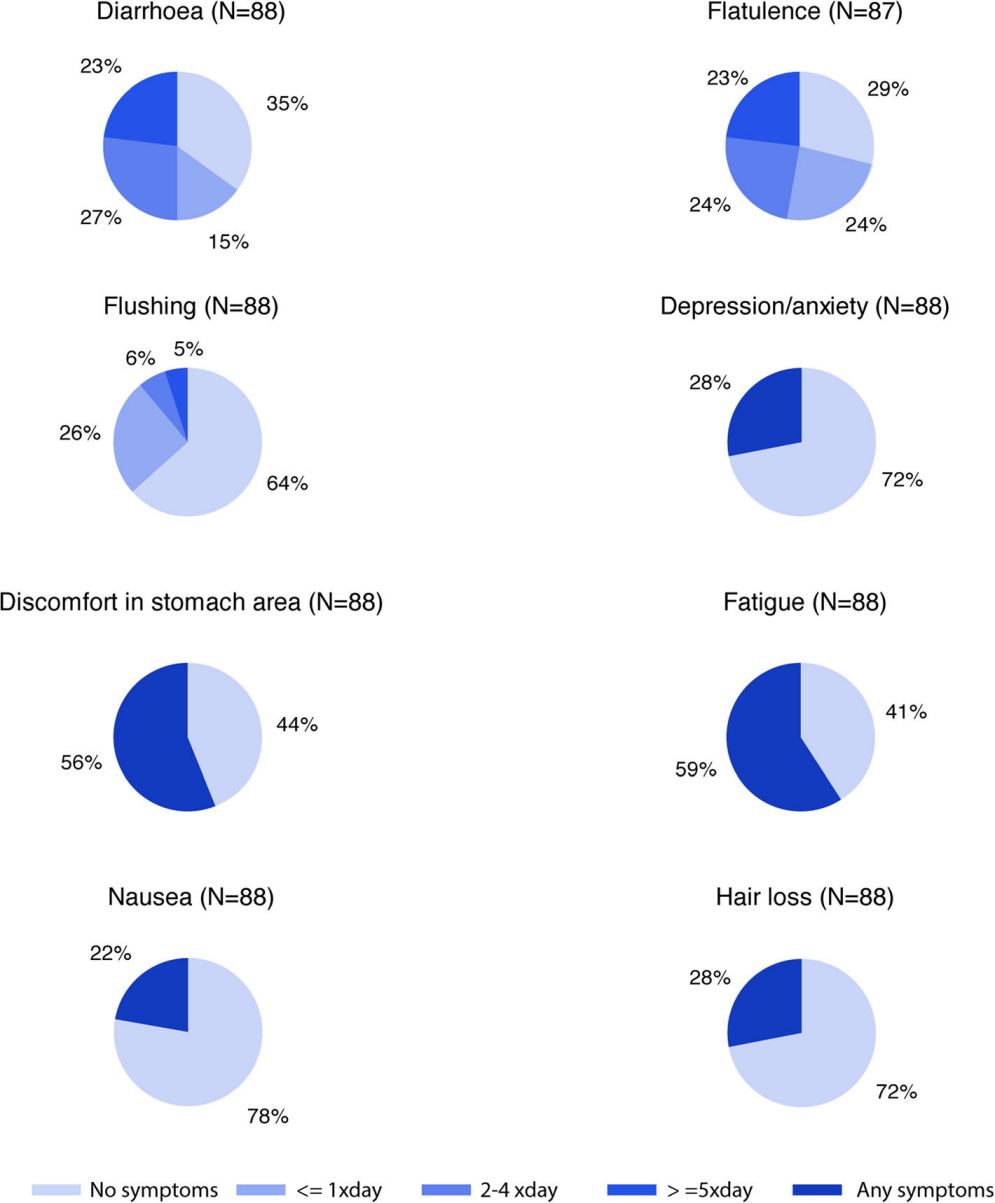
Interview by study nurse: Symptoms in patients on somatostatin analogue treatment for metastatic disease from small-intestinal neuroendocrine tumour at baseline

**Fig. 2:**
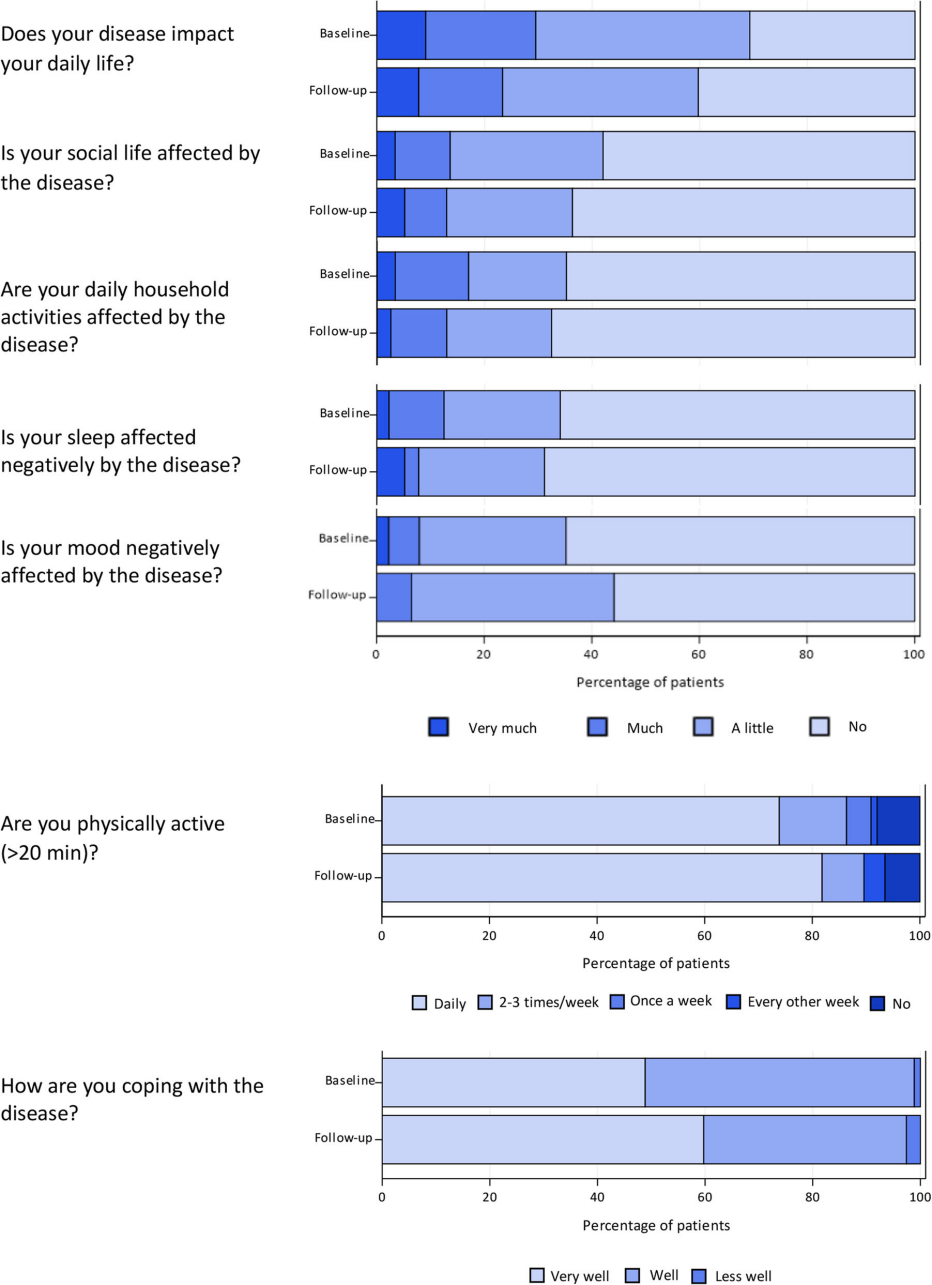
Interview by study nurse: Impact on everyday life and coping with the disease in patients on somatostatin analogue treatment for metastatic disease from small-intestinal neuroendocrine tumour at baseline and at 1-year follow-up

In the nurse interviews, approximately half of the patients reported immediate and persistent symptoms from the injections both at baseline and at the 1-year follow up (Table 4). More than half of the patients reported flatulence (72%), diarrhoea (65%), fatigue (59%) and abdominal discomfort (56%) at baseline (Fig. 1). The results at the 1-year follow-up showed only some minor improvements (Table 4). More than 20% had diarrhoea or flatulence > 5 times per day. During the interview, the patients were asked which symptom they regarded as most troublesome. More than one third of the patients (39%) answered diarrhoea, 16% answered fatigue. Abdominal pain and flatulence were the main complaint for 11% of patients, respectively. For all other symptoms, less than 5% of the patients reported them as their main complaint and 18% of the patients had no major complaint. A comparison between the symptoms reported at baseline by patients on treatment with lanreotide (*n* = 34) versus patients on treatment with octreotide LAR (*n* = 54) is shown in Table 4. There were no major clinically significant differences in the proportions reporting any of the symptoms between the two SSA types. Patients were also asked about how their illness affected their everyday life (Fig. 2). In general, patients reported that they coped well or very well with their disease. Only a minor proportion of the patients stated that their sleep was very much or much affected by their disease and a vast majority of patients were physically active (> 20 min) every day.

**Table 4:** Symptoms based on interview by study nurse in patients by somatostatin analogue treatment for metastatic disease from small intestinal neuroendocrine tumour

	Baseline			1-year follow-up
	Lanreotide (***N*** = 34)	Octreotide LAR (***N*** = 54)	Total ***N*** = 88	Total ***N*** = 77
**Acute complaints from the injections,*n*(%)**				
**None**	17 (50.0%)	24 (44.4%)	41 (46.6%)	44 (57.1%)
**Sore lumps beneath the skin**	11 (32.4%)	14 (25.9%)	25 (28.4%)	22 (28.6%)
**Pain**	2 (5.9%)	5 (9.3%)	7 (8.0%)	8 (10.4%)
**Other**	5 (14.7%)	16 (29.6%)	21 (23.8%)	9 (11.7%)
**Persistent complaints from the injections,*n*(%)**				
**None**	20 (58.8%)	34 (63.0%)	54 (61.4%)	53 (68.8%)
**Sore lumps beneath the skin**	6 (17.6%)	7 (13.0%)	13 (14.8%)	15 (19.5%)
**Pain**	1 (2.9%)	3 (5.6%)	4 (4.5%)	2 (2.6%)
**Flatulence**	4 (11.8%)	6 (11.1%)	10 (11.4%)	3 (3.9%)
**Diarrhoea**	5 (14.7%)	5 (9.3%)	10 (11.4%)	2 (2.6%)
**Other**	6 (17.6%)	7 (13.0%)	13 (14.8%)	6 (7.8%)
**Symptoms (based on interview)**				
**Flatulence**	**26 (76.5%)**	**37 (68.5%)**	**63 (71.6%)**	**54 (71.1%)**
*≥ 5 / day (% of total)*	*10 (29.4%)*	*10 (18.9%)*	*20 (23.0%)*	*14 (18.4%)*
*2–4 / day (% of total)*	*9 (26.5%)*	*12 (22.6%)*	*21 (24.1%)*	*26 (34.2%)*
*1–2 / day (% of total)*	*7 (20.6%)*	*14 (26.4%)*	*21 (24.1%)*	*14 (18.4%)*
**Diarrhoea**	**24 (70.6%)**	**33 (61.1%)**	**57 (64.8%)**	**49 (64.5%)**
*≥ 5 / day (% of total)*	*6 (17.6%)*	*14 (25.9%)*	*20 (22.7%)*	*14 (18.7%)*
*2–4 / day (% of total)*	*14 (41.2%)*	*10 (18.5%)*	*24 (27.3%)*	*22 (29.3%)*
*1–2 / day (% of total)*	*4 (11.8%)*	*9 (16.7%)*	*13 (14.8%)*	*12 (16.0%)*
**Fatigue**	**19 (55.9%)**	**33 (61.1%)**	**52 (59.1%)**	**49 (63.6%)**
**Abdominal discomfort**	**21 (61.8%)**	**28 (51.9%)**	**49 (55.7%)**	**37 (48.1%)**
**Flushing**	**12 (35.3%)**	**20 (37.0%)**	**32 (36.3%)**	**32 (41.6%)**
*≥ 5 / day (% of total)*	*1 (2.9%)*	*3 (5.6%)*	*4 (4.5%)*	*2 (2.6%)*
*2–4 / day (% of total)*	*0*	*5 (9.3%)*	*5 (5.7%)*	*6 (7.9%)*
*1–2 / day (% of total)*	*11 (32.4%)*	*12 (22.2%)*	*23 (26.1%)*	*23 (30.3%)*
**Depression/anxiety**	**7 (20.6%)**	**18 (33.3%)**	**25 (28.4%)**	**26 (33.8%)**
**Partial hair loss**	**10 (29.4%)**	**15 (27.8%)**	**25 (28.4%)**	**22 (28.6%)**
**Nausea**	**7 (20.6%)**	**12 (22.2%)**	**19 (21.6%)**	**12 (15.6%)**
**Effects of diet**				
**Reaction to certain food items**	22 (64.7%)	33 (61.1%)	**55 (62.5%)**	**47 (61.0%)**
**Diarrhoea**	15 (44.1%)	24 (44.4%)	39 (44.3%)	34 (44.2%)
**Flatulence**	16 (47.1%)	22 (40.7%)	38 (43.2%)	30 (39.0%)
**Flushing**	2 (5.9%)	2 (3.7%)	4 (4.5%)	4 (5.2%)
**Other**	6 (17.6%)	9 (16.7%)	15 (17.0%)	12 (15.6%)
**Needs to avoid certain food**	13 (38.2%)	22 (40.7%)	**35 (39.8%)**	**29 (37.7%)**
**Reacts to alcohol**	11 (32.4%)	19 (35.8%)	**30 (34.5%)**	**24 (32.0%)**

### Vitamin status

At baseline, levels of vitamin D were below the recommended value in 24 (27%) patients. For vitamin A, vitamin K and vitamin E, the number of patients with low values were 11 (13%), 6 (13%) and 2 (2%) respectively. At the 1-year follow-up, similar results were reported. Two-thirds of patients (67%) reported taking vitamin supplements at the baseline visit (Table 1). In the subgroup of patients who took vitamin supplements, the levels of vitamin D and A were below normal in approximately 25% and 15% of patients respectively, and did not differ from patients not taking vitamin supplements.

## Discussion

Although the literature on NET in general is extensive in terms of epidemiology, pathophysiology and prognosis, there are few high-quality studies of self-reported symptoms, symptomatic treatment outcomes and quality of life in patients with metastatic small-intestinal NET on long-term treatment with SSA [18]. By using both well-validated questionnaires and a structural interview we wanted to systematically record symptoms and general well-being in these patients. We found that a high proportion of the patients on long-term SSA treatment reported severe symptoms. More than half of the patients reported flatulence (72%), diarrhoea (65%), fatigue (59%) and abdominal discomfort (56%). Some patients had quite severe symptoms as more than 20% had diarrhoea or flatulence more than 5 times per day. Despite this they reported a high quality of life and they coped with their symptoms and managed their daily life well. Despite the number and severity of symptoms reported by the patients, 43% of the patients of working age were employed, not far from the overall 60% employment proportion of Norwegians aged 60–66 years [19].

Although the patients belonged to specialised NET centres and had been under care with SSA for a median of 3 years, the rate of symptoms was high. This may indicate that the attention given to these symptoms from health care workers might be sub-optimal. These patients are usually seen every 6 months at follow-up and then the focus often is on possible tumour progression. The use of a short well-structured follow-up questionnaire on symptoms and a systematic approach to symptom treatment at every follow-up could therefore be useful. An alternative explanation on the frequent symptom burden is that many of these symptoms might be difficult to improve. Several drugs such as pancreatic enzymes, loperamide and cholestyramine are commonly prescribed to improve symptoms. In our study, relatively few patients used anti-diarrhoeal medication (23%) or pancreatic enzyme supplements (30%) compared to the symptom burden the patient cohort had. There are limited data on the true benefit of these drugs on symptoms in NET patients and collecting such data prospectively would be useful. Our study indicates a need for new symptomatic treatment options and an evaluation of their usefulness. Telotristat indicated for diarrhoea in NET patients was not approved during the study period and might have been beneficial for some of these patients [20]. A novel observation in our study was that 63% had reactions to certain food, 40% had to avoid certain food and 35% reacted to alcohol. Although listed as a rare side effect to SSA, 29% complained of partial hair loss. As many as 20% of the patients complained of persistent sore lumps at injection sites. We found no clinically significant differences in side-effects between the two SSA drugs. We found only minor improvements in the patients’ symptoms and general well-being from baseline to the 1-year follow-up. Since most of the patients had been using SSA for years (median 3.1 years) and the median time since their NET diagnosis was almost 5 years, this indicates that they were mainly patients with stable disease who were considered to have effect of SSA and had probably reached an optimal dose. The lack of major improvements of symptoms during the 1-year follow-up might be due to already having tried most of the available symptomatic treatments. The participation in the study with extra appointments with the opportunity to present and discuss their symptoms and QOL with an expert clinical nurse, could be a reason for a better functional score after 1 year. Improvement in quality of life has mainly been seen in patients in earlier years of receiving SSA treatment [21]. Due to the slow-growing behaviour of small-intestinal NET, and to the tumour stabilising effect of SSA, a significant deterioration in their health condition that would have an impact on the results from the 1-year follow-up was not to be expected. Regarding symptoms registered in the questionnaires and interviews, we found a good correlation with diarrhoea, fatigue and pain being the most distressing symptoms. This indicates that questionnaires could be used in our patient population to register the main symptoms experienced by NET patients using SSA. Relative similar findings at 1 year illustrates that the reported symptoms reflected well the continuous everyday symptoms of these patients.

It is difficult to find other studies for comparison of our results. In published quality of life studies on NET, the study populations are frequently heterogeneous, the methodology differs and the quality of the data handling and reporting is variable [18]. Published studies with comparable patient cohorts to ours tend to be interventional studies where only the differences between groups are presented [22, 23], the data are transformed [23], the data presented only summarily [24], the data presented in only figures with low resolution [25] or other questionnaires are used [26, 27]. Some studies include only patients with progressive disease making it difficult to compare with our patients where the majority had stable disease [28]. The SSA treatment arm of the NETTER-1 trial at baseline, with patients comparable to ours, scores close to our results for EORTC QLQ.C30 and QLQ G.I.NET21 [9]. These patients were all using octreotide LAR and reported fatigue, muscular/bone pain and diarrhoea to be the most prominent symptoms. In a study with lanreotide, the results for the EORTC QLQ.C30 score were also comparable to our results; however diarrhoea was a less prominent symptom in this study population [29]. Recently a US study collected quality of life data through a mobile application in 120 NET patients (61% GI primary) who were using long-acting SSA [30]. The most common symptoms were fatigue (77%), diarrhoea (63%), abdominal discomfort (64%) and trouble sleeping (58%). We found in our study no change in symptom severity over the study period of 1 year. This reproduce the findings in a recently published study were 2.271 NET-patients were followed for 5 years and the symptom load remained almost unchanged over the study period [31].

A substantial proportion of the patients in our study had vitamin deficiencies. Vitamin deficiency in NET patients has been shown in prior studies. Vitamin B3 deficiency was found in 45% of patients with serotonin producing tumours [32] and vitamin D and vitamin B12 deficiencies have been found in patients with small-intestinal NET [14, 33]. Among 35 carcinoid patients on long-term SSA treatment (> 18 months), 80% had deficiencies in fat-soluble vitamins [14]. Similarly to our results, about 30% had vitamin D deficiency, whereas they found a much higher proportion of vitamin K deficiency. In our study a substantial number of patients used vitamin supplements, however, this did not seem to protect against vitamin deficiency. Over the counter vitamin supplements may not contain high enough doses to prevent vitamin deficiency in NET patients. We did not collect data on vitamin preparation and dose, hence, we cannot conclude regarding the optimal does of lipid soluble vitamins these patients should take. Our study supports the ENETS guidelines recommending that vitamin B and fat-soluble vitamins should be monitored in NET patients on SAA treatment [34]. Vitamin D deficiency seems to be frequent both in NET patients [35, 36] and individuals without health problems. In the general Norwegian population, 13.5% have been reported to have mild to moderate Vitamin D deficiency < 25 (OH) D nmol/L [37]. Vitamin D deficiency is implicated in the aetiology of several diseases, including cancer [38, 39]. A recent meta-analysis has shown that high levels of circulating 25-hydroxyvitamin D levels in cancer patients are associated with a significant reduction in both disease progression and risk of death [40]. Vitamin D supplements however do not seem to prolong relapse-free-survival in digestive tract cancers [41].

An interesting finding of the study was that though these patients report a high symptom burden, they also reported a high quality of life and coped well, arguing against a direct correlation between symptom burden and QOL. This speaks to the potential for human adaptation and resiliency even when living with an incurable disease. As many as 90% of the patients knew that they would eventually die from their disease, and 92% knew that the main indication of the SAA treatment was to live longer but with no chance for cure. This is quite in contrast with previous observations in cancer patients before the immunotherapy era. In a study of patients with metastatic lung or colorectal cancer given palliative chemotherapy, 69% of lung cancer patients and 81% of colorectal cancer patients did not report understanding that chemotherapy was not likely at all to cure their cancer [42].

A limitation of our study is that we could not discriminate whether symptoms were post-operative, due to the SSA treatment or the NET disease itself. To better understand the pathophysiology and to offer specific treatments, such discrimination would probably be necessary. To address this issue, symptoms should be recorded prospectively before start of SSA treatment or any surgical procedures and then regularly thereafter. The aim of this study was not to compare the NET population to the general population. We already know that the NET population has a lower health related quality of life compared to the general Norwegian population [13]. It would have been useful to know the level of diarrhoea, pain and fatigue in a comparable background population as these symptoms are non-specific and could arise in several conditions not related to NET. Most comparable studies report only findings in patients and do not have healthy control groups. Furthermore, the patients’ symptoms need care regardless of the possible background incidence in the general population. The cross-sectional design of this study probably leads to inclusion of more patients with a better prognosis using SSA for a longer period. However the target population for this study was patients on SSA treatment for a considerable number of years.

## Conclusions

Overall, it can be concluded that patients with metastatic small-intestinal NET have a high symptom burden during long-term treatment with SSA, but despite this maintain a high quality of life. To further improve NET patient care, it may be beneficial to regularly use short questionnaires for collection of symptoms during SSA treatment. It will be important to try to discriminate between symptoms related to prior surgical procedures, the ongoing SSA treatment or from the NET disease itself to optimise the care. Many of these patients may need differentiated management strategies and a closer structured follow-up to achieve optimal care. Future studies are needed to explore how these symptoms can be managed in a more effective way to further improve the care and ensure that quality of life is as good as possible in each patient.

## Data Availability

The datasets analysed during this study are available from the corresponding author on reasonable request.
